# Coping styles mediate the relation between mindset and academic resilience in adolescents during the COVID-19 pandemic: a randomized controlled trial

**DOI:** 10.1038/s41598-023-33392-9

**Published:** 2023-04-13

**Authors:** T. W. P. Janssen, N. van Atteveldt

**Affiliations:** Department of Clinical, Neuro- & Developmental Psychology & Research Institute Learn!, Faculty of Behavioural and Movement Sciences, Vrije Universiteit Amsterdam, Van Der Boechorststraat 7, 1081 BT Amsterdam, The Netherlands

**Keywords:** Human behaviour, Stress and resilience

## Abstract

The COVID-19 pandemic negatively impacted adolescent mental health on a global scale. However, many students were resilient during this crisis, despite exposure to COVID-related stressors. We aimed to study the protective effects of growth mindset on school-related resilience during the COVID-19 pandemic, and the mediating effects of coping styles. The two-year follow-up of an ongoing Randomized Controlled Trial, involving a growth mindset and control intervention, took place during the pandemic. We measured growth mindset, school burnout symptoms, COVID-19-specific stressor exposure, coping styles, and calculated a resilience score (corrected for pre-pandemic school burnout symptoms). Mediation analyses were performed in the total sample (*N* = 261), and exploratory in the intervention subsamples, to test whether the associations between mindset and resilience were mediated by coping styles. Growth-mindset students were more resilient during the pandemic and used less maladaptive and more adaptive (acceptance) coping styles. Coping mediated the relation between mindset and resilience in the total sample (both coping styles), and growth mindset intervention subsample (maladaptive coping). We found unique evidence for the beneficial effects of growth mindset on school-related resilience during the pandemic, and the mediating effect of coping styles as explanatory mechanism. This work contributes to a growing literature that shows positive effects of growth mindset on mental health.

## Introduction

The COVID-19 pandemic impacted adolescent mental health on a global scale^1^. Closures of schools and other learning spaces have impacted 94 per cent of the world’s student population^2^, resulting in increased social isolation^3^, and more challenging learning conditions^4^. The negative consequences of social isolation may have a greater impact on adolescents and young adults^5,6^, as social interactions with peers are important for identity development and support^7,8^. This is particularly worrisome, considering that the peak onset of mental health disorders is during adolescence (~14 years)^9^, with potential lifetime vulnerabilities for mental health problems^10^. In the Netherlands, schools first closed down on March 16th 2020 for eleven weeks, followed by a period of partial re-opening, and another full closure for 11 weeks between December 15th 2020 and March 3rd 2021, followed again by partial re-opening. The Netherlands Youth Institute (NJi) and the Dutch Inspectorate of Education published worrying reports about the damaging effects on mental health, motivation for learning, socioemotional development^11,12^ and academic performance^13^, in line with international research^1^.

However, a considerable subgroup of students did relatively well despite the pandemic^12,14,15^. Whether or not students were resilient during this crisis—maintaining mental health despite stressor exposure^16^—seems to be influenced by well-known risk and protective factors, such as socioeconomic status, social support and coping skills^1^. Especially interesting from a prevention perspective, are a special kind of protective factors called ‘resilience factors’; these are relatively stable predispositions that increase the likelihood of resilient responses to stressors, such as emotion regulation capacity, communication ability and positive appraisal style^16,17^. One potential resilience factor—growth mindset—is especially relevant in an educational context^18–21^ and suitable as a preventive target to reduce the impact of future virus variants or pandemics on adolescents’ school-related mental health.

Mindset theory^22^ posits that students hold varying implicit beliefs about the malleability of personal attributes, such as intelligence or math ability. These beliefs range on a continuum, with some students holding entity beliefs (or a fixed mindset), believing that intelligence is relatively stable, while others holding incremental beliefs (or a growth mindset), believing that intelligence can be improved through their own efforts. These beliefs in turn affect self-regulation, academic achievement and motivation. Generally, students with a growth mindset exhibit adaptive self-regulation skills, in which they set learning goals (rather than performance goals), adopt mastery-oriented strategies when encountering setbacks (rather than helpless-oriented strategies), and focus on future expectations of success (rather than negative emotions)^23^. These adaptive self-regulation skills are conducive for more resilient responses during challenging times^18,19^. Therefore, we predict that students with a growth mindset maintain better school-related mental health in the current pandemic.

Recently, there has been a growing interest in the association between mindset and mental health. In their meta-analysis, Burnette et al.^20^ found that growth mindset was related to less distress, more active coping and higher value placed on treatment. In a subset of eight studies where mindset was experimentally manipulated, more encouraging effect sizes were observed for mental health outcomes when compared to academic outcomes^24^. Mindset has also been associated with school-related mental health. Cross-sectional evidence demonstrated a negative relation between growth mindset and school burnout symptoms^25^. School burnout symptoms, defined as school-related exhaustion, cynicism, and sense of inadequacy^26^, may be important indicators of mental health problems in vulnerable students. Two other cross-sectional studies explored how mindset was related to school-related mental health during COVID-19. Zhao et al.^27^ found that the positive relation between growth mindset and learning engagement in undergraduates, was mediated by reduced perceived COVID-19 strength and perceived stress. In line with these results, Mosanya et al.^28^ found a negative association between growth mindset and academic stress during the pandemic. Interestingly, Yeager et al.^18^ reported reduced generalized anxiety symptoms during the pandemic, only for students with negative prior mindsets (interpreting stressful events and responses as harmful/uncontrollable) who received a synergistic mindset intervention before the pandemic.

Based on the discussed literature, students with a growth mindset may employ more adaptive coping styles (e.g., active coping) and less maladaptive coping styles (e.g., behavioural disengagement) to deal with COVID-19-related challenges, and therefore are more resilient in the school context. Coping is defined as cognitive and behavioural strategies that individuals apply to manage demands evaluated as taxing^29^, with theories of coping being classified according to orientation (situational versus dispositional) and approach (macro or microanalytic)^30^. One of the most frequently used questionnaires to measure coping is the brief COPE (Coping Orientation to Problems Experienced)^31^, which has a microanalytic approach with 14 subscales. Depending on its framing, it can be used to measure either situational or dispositional coping. Theoretically, the brief COPE was based on *problem-focused, emotion-focused* and *less useful coping strategies*^32^. However, a recent review of 85 studies revealed highly inconsistent ‘macro-analytic’ factor structures, ranging between 2 and 15 factors (with a mode of 2 factors)^30^. Dichotomies such as approach/avoidant, adaptive/maladaptive and active/disengaged were frequently reported, while among all studies, disengaged/active/social support coping were most consistently identified. Various factor structures have also been used for the brief COPE during the COVID-19 pandemic^32–34^.

The current literature has several limitations that need to be addressed. First, most COVID-19-related research, for understandable reasons, lack a pre-pandemic assessment of (school-related) mental health, limiting the potential to isolate COVID-specific effects (for notable exceptions, see^6,15^). Second, even more rare are studies that manipulate resilience factors before the pandemic, such as growth mindset, with a randomized controlled (RCT) design and follow-up during the pandemic. This could provide even more compelling causal evidence. Third, most resilience research does not take into account the actual exposure to adversity, but rather rely on resilience questionnaires^35^ that are conducted before any adversity has occurred^16^. When considering resilience as an outcome—maintaining mental health despite stressor exposure—it follows that adversity needs to occur and be measured as well. Fourth, few studies have focused on school-related mental health, while this can have important consequences for learning outcomes and later societal opportunities.

**Figure 1:**
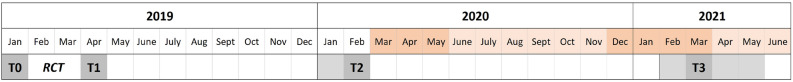
Timeline of the study in relation to the COVID-19 pandemic. Note. This timeline shows the timing of the study (grey) in relation to the COVID-19 pandemic (orange; dark orange depicts full school closures, while light orange depicts partial school closures). Schools first closed down on March 16th 2020. T0 (baseline), RCT (randomized controlled trial; adolescents either received a growth mindset intervention or control intervention), T1 (direct-post), T2 (1-year follow-up; pre-pandemic) and T3 (2-year follow-up; peri-pandemic). Dark grey depicts the mean date questionnaires were received, light grey depicts the entire time period in which questionnaires were received. Resilience, coping and mindset were measured at T3. Resilience was defined as residuals of the regression between school burnout symptoms and COVID-related stress exposure, controlled for burnout at T2 (normative modelling method). Coping was measured based on ‘the last year, since the pandemic started’.

By coincidence, our research group was conducting an RCT study into a growth mindset intervention for high-school students and just finished the one-year follow-up measurement, when the pandemic hit (see Fig. 1 for the timeline). Given this unique situation, we decided to make several amendments to the two-year follow-up, with the aim to study how mindset relates to school-related resilience during the COVID-19 pandemic, and to investigate the mediating role of coping styles. An additional aim was to explore the effects of the growth mindset intervention on this mediation. Because we were interested in resilience as an outcome—maintaining mental health despite stressor exposure—we needed to measure both school-related *mental health* (school burnout symptoms) and *exposure* to stressors specific for the COVID-19 crisis^17^. One would expect that students with higher exposure, also report more burnout symptoms. A more resilient student, however, will report lower burnout symptoms than expected, given the stressor exposure. We quantified this as a residual onto the regression curve between exposure and burnout symptoms^36^. The pre-pandemic measure of school burnout allowed us to correct for differences that were unrelated to the pandemic. Finally, we added the brief COPE^31^ to measure situational coping styles that students have used since the beginning of the pandemic. Given that the COVID-19 pandemic has different characteristics than disasters that are usually studied in coping research^6^, and the inconsistency about higher-order coping styles in past research, we used a data-driven approach to determine higher-order coping styles in the current study. Growth mindset is expected to be related to active coping^20^, in accordance with more adaptive self-regulation strategies that are characteristic for growth-minded students^23^. In contrast, fixed mindset is expected to be related to maladaptive coping styles, in accordance with helpless-oriented strategies that are characteristic for fixed-minded students^23^.

Based on previous research showing that differences between fixed and growth mindset are amplified by set-backs^23^ and challenging transitions^37^, we hypothesized that (1a) students with a growth mindset would be more resilient during the pandemic, and (1b) report more adaptive and less maladaptive coping styles, (2) the relation between mindset and resilience would be mediated by coping styles, where students with a growth mindset would employ more adaptive/less maladaptive coping styles, which in turn would relate to higher resilience, and (3) exploratively, we repeated the mediation analyses separately for those who received either a growth mindset or control intervention one year before the pandemic started.

## Methods

### Trial design

The reporting of this study follows the CONSORT-SPI 2018 guidelines for social and psychological interventions with cluster extension^38^, see Supplementary Table S1 online. This study is part of a larger parallel cluster randomized controlled trial (RCT), stratified for school (n = 2) and educational track in the Dutch secondary school system (n = 5: ranging from vocational to pre-university). With an allocation ratio of 1/1, twenty 7th grade classes were randomized to either the experimental growth mindset intervention or control intervention. Outcomes were evaluated before (T0), directly after as manipulation check (T1), 1 year later (T2) and 2 years later (T3). The main results of this RCT are reported elsewhere^39^, which focus on intervention-related improvements in growth mindset and academic achievement between T0-T2. The current study, however, focuses only on T2 (pre-pandemic) and T3 (during the pandemic: i.e., peri-pandemic) data, to test the COVID-related hypotheses.

This trial was preregistered at the Dutch Trial Register (04/03/2019, Trial NL7562), approved by the local ethics committee (Vaste Commissie Wetenschap en Ethiek; VCWE-S-18-00149) and conducted in accordance with the Declaration of Helsinki. We made a few unforeseen changes following trial commencement. The COVID-19 pandemic started in between T2 and T3. We added two new questionnaires (T3) with the aim to investigate the relation between mindset and resilience during COVID-19, and the mediating role of coping mechanisms: brief Cope^31^, COVID-related stress Exposure^17^. For these additional questionnaires, an amendment was approved by the local ethics committee (VCWE-S-21-00041).

### Randomization

Randomization of classes (cluster allocation) was realized using the RANDARRAY function in excel, which generated random numbers between 0 and 1, with 15 decimal places. For each of the two schools, a random number array was generated for the participating classes within each school (n = 8, n = 12). Because we used stratification for both school and educational track, with the additional restriction of even frequencies within each educational track, we could make duo’s of two classes within the same educational track of the same school. The class with the highest random number was allocated to the growth mindset intervention, while the class with lowest random number was allocated to the control intervention. All participants within each class were invited to participate, see Participants.

The following order of events was implemented in this RCT: enrollment of schools, enrollment of classes, consent of individual students, random allocation, start interventions. Allocation was concealed throughout the RCT for participating students, parents and class mentors, but not the researchers. Although we did not explicitly mention the allocation, it was possible for participants to identify the allocated intervention, based on the participant information they received during enrollment. However, this information only described the interventions as ‘*two different courses about the brain*’ focusing either on ‘*Opportunities and myths*’ (control intervention) or ‘*Plasticity*’ (growth mindset intervention), concealing the actual aims of the experimental intervention (stimulating a growth mindset). The lead investigator, T.W.P. Janssen, generated the random allocation sequence, enrolled the classes and assigned clusters to the interventions.

### Participants

Two urban schools in the West of the Netherlands participated in this study with a total of 20 classes. All students in these 20 classes were asked to participate in the study (n = 553), with no exclusion criteria to allow stronger generalizability of findings. Parents of eligible students received a flyer and additional information about the study. A sample of 439 students (79%) decided to participate, with both parents and students giving active informed consent, of which 426 students returned the T0 questionnaire.

T3 data were available for 289 students (68% of *N* = 426 at T0), while complete data for both T2 and T3 were available for 271 students (64%). Ten participants filled in the T2 questionnaire *after* schools first closed down on March 16th 2020, which therefore had to be excluded as T2 was a pre-pandemic measure, resulting in a final sample of 261 students (61%; T2 completed between January and March 16th 2020). T3 questionnaires were returned between 11-02-2021 and 03-05-2021, with a mean of 09-03-2021 and a median of 17-03-2021, see Fig. 1. We performed an attrition analysis to explore whether this sample (*N* = 261) is comparable to those from the T0 sample who could not be included (*N* = 178) on several group characteristics at T0. There were no statistical differences in age, *F*(1,424) = 1.63, *p* = 0.203, socio-economic status, *F*(1,434) = 0.05, *p* = 0.825, growth mindset, *F*(1,424) = 0.82, *p* = 0.367, and gender, χ^2^ = 0.29 (1), *p* = 0.593, indicating comparability.

### Interventions

The following paragraphs contain brief descriptions of both intervention conditions, but see the published results^39^ and the archived data for all intervention materials translated in English (stored at DataverseNL). The control and growth mindset intervention conditions were matched on duration (4 lessons × 50 min) and frequency (1 per week). While both intervention conditions were about exploring your own brain, the key difference was that only the growth mindset intervention focused on brain plasticity and controllability, mindset, and their interrelatedness.

#### Control intervention

Students learned about brain anatomy (*lesson 1*), brain illusions (*lesson 2*), brain imaging techniques (*lesson 3*) and brain myths and opportunities (*lesson 4*). Most exercises involved active participation, such as making a brain-hat (lesson 1), discussing various visual illusions, including ones in real-life (lesson 2), seeing, but not influencing, their own brain waves measured with mobile EEG (lesson 3), and participating in a neuromyth quiz and mailing a postcard to their future selves (lesson 4).

#### Growth mindset intervention

Students learned about brain plasticity (*lesson 1*), growth-mindset (*lesson 2*), experienced influence over their own brain activity using mobile EEG-based neurofeedback (*lesson 3*), and learned how lesson 1–3 were related to their own school career (*lesson 4*). Most exercises involved active participation, such as performing a mirror-drawing task (lesson 1), reflecting on former fixed and growth-mindset reactions to challenging events at school (lesson 2), influencing a brain correlate of focused attention (theta/beta index) with mobile EEG neurofeedback (NFB; lesson 3) and formulating SMART goals (Specific, Measurable, Achievable, Relevant, and Time-Bound) to implement a growth-mindset in school and mailing a postcard to their future selves (lesson 4).

All lessons were provided by researchers: eight undergraduates with a (research) master in developmental (neuro)psychology or neuroscience, between 28-01-2019 and 22-02-2019 (school 1) and between 11-03-2019 and 05-04-2019 (school 2), during regular mentor hours. Pairs of undergraduates jointly gave both the control and growth mindset interventions, to eliminate potential teacher biases. Undergraduates were trained and supervised by the lead investigator of this study, T.W.P. Janssen, and followed a detailed protocol for teaching each lesson. There were no statistical differences in registered attendance of participating students between the growth mindset and control groups (means: 3.61 and 3.66 out of four lessons), F(1,437) = 0.398, p = 0.528. Across all participants, 76.8% attended all 4 lessons, and 94.6% attended 3 or 4 lessons.

### Outcomes

The current study addresses the dynamic interplay between mindset, resilience and coping *during* the pandemic (T3). Because previous literature indicated a relationship between mindset and mental health^20^, we controlled for pre-pandemic (T2) school burnout symptoms, to isolate mental health effects specifically related to the pandemic.

#### Mindset (T3)

Mindset was measured using the revised self-theory scale designed by De Castella and Byrne^40^, translated to Dutch^41,42^. This questionnaire consists of eight items. Each item was scored on a Likert scale from 1 (strongly disagree) to 6 (strongly agree). The scores on the four fixed mindset items were reversed and added to the scores of the four growth mindset items to create one mindset scale^41,42^, ranging between 8 and 48. Higher scores reflected greater growth mindset endorsement. An example item for fixed mindset is: “*I don't think I personally can do much to increase my intelligence”.* An example item for growth mindset is: “*I believe I can always substantially improve on my intelligence*”. Internal consistency was good (α = 0.896).

#### School burnout symptoms (T2 and T3)

The nine-item School Burnout Inventory^26^ (SBI; translated to Dutch) was used to measure students’ school burnout symptoms, including three scales: (1) exhaustion at school (four items), (2) cynicism towards the meaning of school (three items) and (3) sense of inadequacy at school (two items). Examples of items for each subscale are respectively “*I feel overwhelmed by my schoolwork*”, “*I feel that I'm losing interest in my schoolwork*”, and “*I often have feelings of inadequacy in my schoolwork*”. The extent to which students agreed with the statements were indicated on a six-point Likert scale (1 = Totally disagree to 6 = Totally agree). A total score^26^ was computed (ranging between 9 and 54), in which a higher score indicated that students experienced more school burnout symptoms. Internal consistency was good (T2, α = 0.839; T3, α = 0.880).

#### Coping styles (T3)

Coping was measured using the brief COPE^31^ (translated to Dutch), consisting of 28 items. The brief COPE has been successfully used in adolescents in previous studies^43–49^. We excluded two substance use items, based on very low percentages of moderate or high use (~ 3%), indicating low suitability for this age group. Students received the following instruction to measure situational coping: “*The following questions ask how you have tried to cope with the COVID pandemic. Read the statements and indicate how much you have been using each coping style the last year, since the pandemic started*”. Each item was scored on a Likert scale from 1 (strongly disagree) to 4 (strongly agree).

We applied Principal Component Analysis (PCA) in SPSS 26.0 to extract higher-order coping factors. The sample was considered adequate^50^, with a Kaiser–Meyer–Olkin (KMO) of 0.824 and a significant Bartlett’s test (< 0.001). Components with Eigenvalues of over 1 were retained as components. We used oblique rotation (direct oblimin), allowing correlations between factors, which is plausible between coping styles. Any coefficients below 0.4 were excluded. Extraction suggested that 25 items loaded on 6 factors (1 item did not load on any factor), accounting for 59.6% of variance, which is a common amount in humanities (50–60%)^51^. Examination of the scree plot confirmed this solution, with no cross-loadings according to the Pattern Matrix table. After examination of the items loading on the 6 factors, we calculated sum scores for each of the following factors to use in subsequent analyses: (1) Maladaptive (5 items; α = 0.804), (2) Active (8 items; α = 0.803), (3) Social support (4 items; α = 0.847), (4) Religion (2 items; α = 0.783), (5) Acceptance (4 items; α = 0.686), (6) Humor (2 items; α = 0.622). See Supplementary Tables S2 and S3 online for the factor loadings, the scale questions ordered according to the 6 factors, the corresponding original brief COPE scales, and the rationale for the interpretation and naming of each factor.

#### COVID-related stress exposure

This questionnaire was developed by Veer et al.^17^ (translated to Dutch) to assess the exposure to stressors specific for the COVID-19 crisis. As discussed, most resilience research neglects measuring actual exposure to adversity, while this is central to the definition of resilience: maintaining mental health despite stressor exposure. Of the 29 original items, we selected 22 that were relevant for adolescents. Students received the following instruction: “*Following are some situations that people may experience as a result of the current COVID-19 pandemic. Please indicate whether you are currently experiencing the following situations, or have experienced them in the past year, in connection with the COVID-19 pandemic, and how burdensome they are/were for you*”. Each item was scored on a Likert scale from 0 (did not take place) to 5 (very burdensome). Examples are: “*Having COVID-19 symptoms, or symptoms that could be related to COVID-19*”, “*Family, friends, or loved ones being at increased risk for a serious course of the disease in case of an infection (they belong to a so-called 'risk group')*” and “*Loss of social contact*”. We collapsed the scores into counts^17^, with an answer between 1 and 5 indicating the presence of a particular exposure, and an answer of 0 indicating the absence, leading to a sum score between 0 and 22, with higher scores indicating more COVID-related stress exposure. We chose this scoring scheme to arrive at a more objective measure of exposure, by coding only presence versus absence of exposures (discarding the information about burdensomeness), with minimal appraisal^17^.

### Analytical methods

All analyses were performed with SPSS 26.0^52^. First, we will explain how the resilience variable was calculated, which we used as dependent variable in the subsequent analyses. This is followed by explaining the (1) correlational analyses to test hypothesis 1a and 1b (*students with a growth mindset during the pandemic display more resilience, and report more adaptive and less maladaptive coping styles during the pandemic*), and (2) mediation analyses to test hypothesis 2 (*the relation between mindset and resilience is mediated by coping styles, where students with a growth mindset employ more adaptive and less maladaptive coping styles, which in turn relates to higher resilience*), and (3) exploratory mediation analyses separately for those who received either a growth mindset or control intervention two years earlier. We used a p-value of < 0.05 as indicator of significance for all analyses.

### Calculation of resilience variable

**Figure 2:**
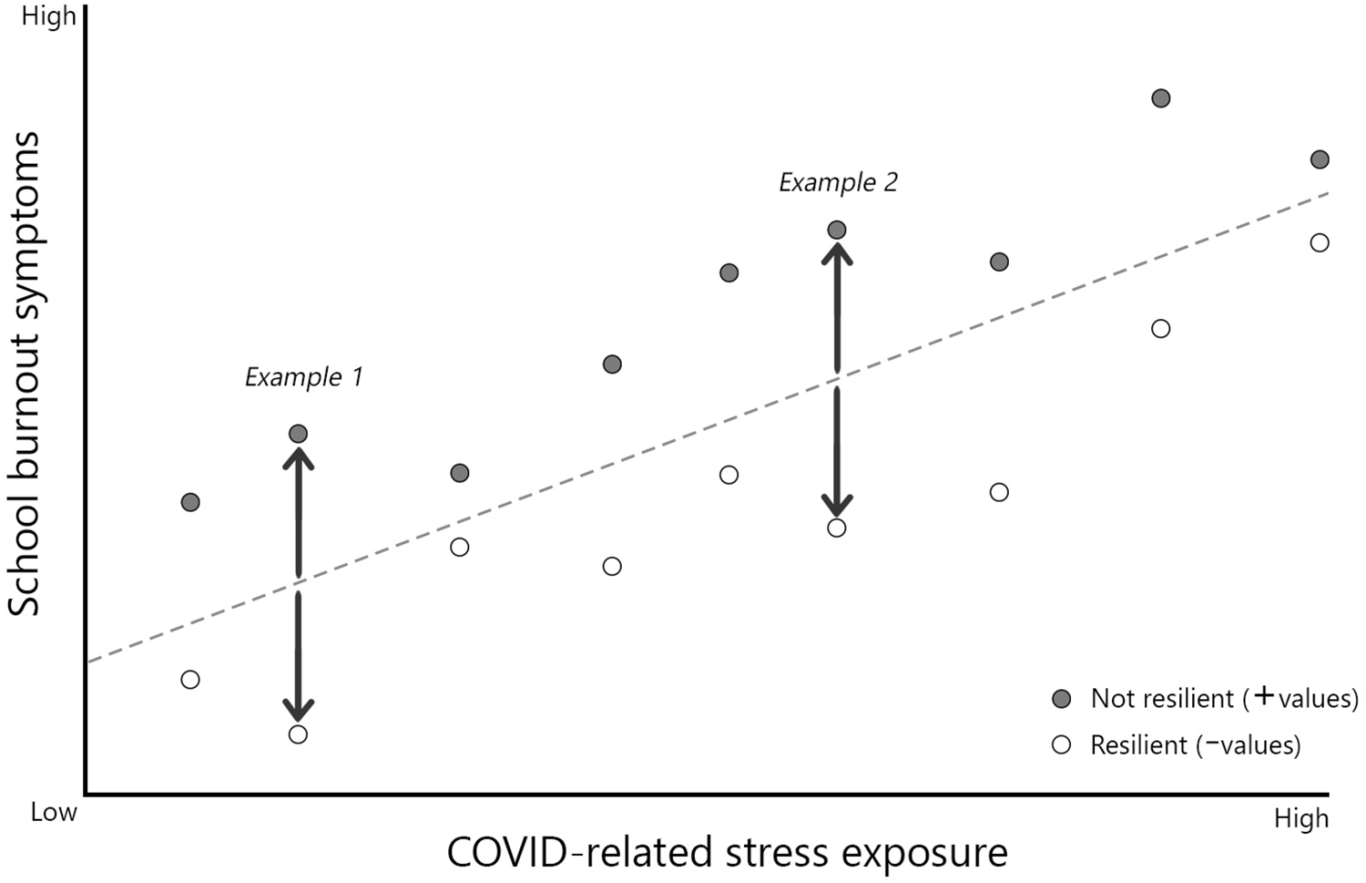
Explanation of resilience variable. *Note.* Resilience is defined as residuals of the regression between school burnout symptoms and COVID-related stress exposure (observed [dots]—predicted value [regression line]), depicted here as the distance between the regression line and observed values. This is a normative modelling method, as it inherently corrects for individual differences in stressor exposure^17^, which is demonstrated with examples 1 and 2 that have identical residuals. Negative values (open circles) are resilient students, as they maintain better school-related mental health (lower school burnout symptoms) than one would predict based on their level of COVID-related stress exposure.

A hierarchical multiple regression was conducted with school burnout symptoms at T3 (during the pandemic) as dependent variable (Y). We first included school burnout symptoms at T2 (pre-pandemic) as independent control variable (Model 1), and then also included COVID-related stress exposure (Model 2). The residuals of Model 2 (observed minus predicted value) were used in further analyses as resilience variable, based on^16^, with negative values indicating more resilient outcomes (*fewer* school burnout symptoms than expected given stress exposure) and positive values indicating less resilient outcomes (*more* school burnout symptoms than expected given stress exposure). This is a normative modelling method, as it inherently corrects for individual differences in stressor exposure^17^. See Fig. 2 for a visual explanation.

### Correlation and mediation analyses

We first calculated Pearson correlations between mindset, coping styles (based on PCA), and resilience (resilience variable as explained above). In the case of significant relations between X (mindset at T3) and the mediator M (coping styles at T3), and between M and Y (resilience at T3, controlled for T2), we decided to proceed with mediation analyses^53^.

Mediation analyses were performed with the PROCESS (version 3.5.3) plugin for SPSS^54^. Mindset was the independent variable (X), coping style the mediator variable (M), and resilience was the dependent variable (Y). Mediation analyses were performed separately for each coping style that was significantly related to both mindset and resilience. Indirect effects were evaluated based on 95% confidence intervals constructed using a bootstrapping procedure with 5000 samples, where a confidence interval entirely above or below zero demonstrates a significant effect. Unstandardized regression coefficients (b) are reported. Based on power analyses conducted by Fritz & MacKinnon^53^, a sample size of 162 is required to find a significant mediation effect with a power of 0.80, using the percentile bootstrap method, and both *a* path and *b* path effect sizes between small and medium. With a sample of 261, it is therefore possible to demonstrate small-medium effects. In a next exploratory step, the mediation analyses were performed separately for the control intervention (n = 130) and growth mindset intervention (n = 131) groups. Based on power analyses conducted by Fritz & MacKinnon^53^, a sample size of 126 is required to find a significant mediation effect with a power of 0.80, using the percentile bootstrap method, with one of the *a* or *b* paths having an effect size of small-medium and the other medium. With the subsamples, it is therefore possible to demonstrate small-medium and medium effects.

## Results

### Descriptive Statistics

For means, *SD* and minimal and maximum scores, see Table 1. To test the hypotheses, results will be presented in the following order: (1) calculation of the resilience variable, (2) correlations between mindset, coping styles (based on PCA, see Methods and Supplementary Tables S2 and S3 online), and resilience, (3) mediation analyses.

**Table 1:** Participant characteristics.

	*N* = 261		
	Mean (*SD*)	Minimum	Maximum
Demographic data			
Age T3 (years)	14.82 (0.47)	13.09	16.21
Gender (M/F)	134/127		
Questionnaires			
Growth mindset T3	35.08 (6.61)	12.00	48.00
School burnout T2	30.73 (8.20)	12.00	53.00
School burnout T3	32.41 (8.92)	9.00	54.00
COVID exposure T3	13.16 (3.89)	0.00	21.00
*Coping styles (PCA)*			
Maladaptive (5 items)	8.29 (3.23)	5.00	18.00
Active (8 items)	17.60 (4.60)	8.00	29.00
Social support (4 items)	7.91 (2.98)	4.00	16.00
Religion (2 items)	2.78 (1.45)	2.00	8.00
Acceptance (4 items)	13.75 (2.25)	6.00	16.00
Humor (2 items)	3.93 (1.53)	2.00	8.00

T2 = pre-pandemic. T3 = during pandemic. M = male. F = female. *SD* = standard deviation.

### Calculation of resilience variable

Model 2 in Table 2 shows a significant relationship between school burnout symptoms and COVID-related stress exposure during the pandemic, while controlling for pre-pandemic school burnout symptoms, *t* = 2.35, *p* = 0.020. The residuals of Model 2 (observed minus predicted value) were used in further analyses as resilience variable (ranging between -23.40 and 21.05), with negative values indicating more resilient outcomes and positive values indicating less resilient outcomes (controlled for pre-pandemic school burnout symptoms), see Fig. 2 for a visual explanation.

**Table 2:** Hierarchical multiple regression results.

Variable	Model 1			Model 2		
	*B*	β	*SE*	*B*	β	*SE*
Constant	12.22		1.72	9.78		1.99
School Burnout T2	.66***	.604	.05	.62***	.57	.06
COVID exposure T3				.28*	.12	.12
*R*^2^		.36			.37	
*ΔR*^2^					.01*	

*N* = 261*.* * *p* < .05. *** *p* < .001. We examined the relation between school burnout symptoms and COVID-related stress exposure (T3: during the pandemic), while controlling for pre-pandemic school burnout symptoms (T2). The residuals of Model 2 were used as resilience variable.

### Correlations

First we calculated correlations (hypothesis 1a and 1b) between mindset, coping styles and resilience, see Table 3. Results showed that students who more strongly endorsed a growth mindset, reported less maladaptive coping, less humor coping, and more acceptance coping. Students with higher resilience, reported less maladaptive coping, less humor coping, and more acceptance coping. Finally, growth mindset correlated negatively with resilience, *r*(259) = −0.126, *p* = 0.041 (note that the resilience variable is reversed), meaning that students who more strongly endorsed a growth mindset, also demonstrated higher resilience. Based on these correlational results, we proceeded with mediation models including growth mindset (X) and resilience (Y), separately for the three coping styles that demonstrated significant correlations with X and Y (M; maladaptive, acceptance, humor).

**Table 3:** Pearson correlations between mindset and resilience, with 6 coping subscales based on PCA.

N = 261	Maladaptive	Active	Social support	Religion	Acceptance	Humor
Growth mindset	−.250***	.077	−.043	.050	.274***	−.243***
Resilience	.381***	.032	.067	.013	−.185***	.122*

Note that the resilience variable is reversed, meaning that higher resilience is related to less maladaptive, less humor, and more acceptance coping styles. * p < .05, ** < .01, *** < .001.

### Mediation analyses

**Figure 3:**
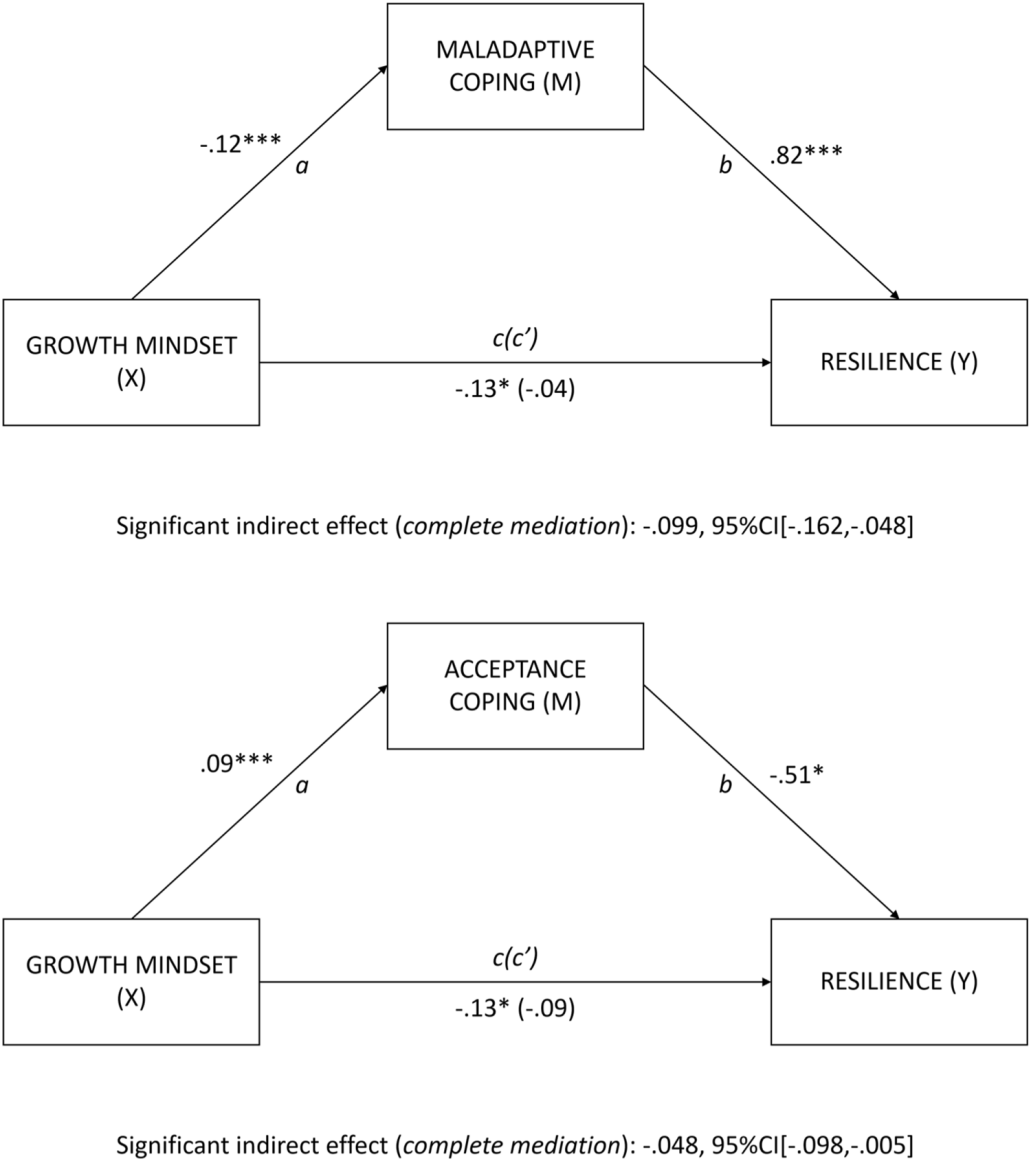
Mediation results: total sample. *Note. N* = 261. X = independent variable. Y = dependent variable. M = mediator. Note, growth mindset and coping styles were measured during the pandemic (T3). The resilience variable was defined as residuals of the regression between school burnout symptoms and COVID-related stress exposure (at T3), controlling for pre-pandemic school burnout symptoms (T2). Note that the resilience variable is reversed. * p < .05, ** < .01, *** < .001; path c’ = direct effect; numbers are beta values.

Maladaptive coping and acceptance coping significantly mediated the relation between growth mindset and resilience, see Fig. 3 (hypothesis 2). For maladaptive coping, there was a significant path *a*, *t*(259) = −4.16, *p* < 0.001, a significant path *b*, *t*(258) = 6.27, *p* < 0.001, and a significant path *c*, *t*(259) = −2.05, *p* = 0.041. There was a significant indirect effect (path a*b) of growth mindset on resilience through maladaptive coping, b = −0.099, Bootstrap CI_95_[−0.162, −0.048], meaning that students who endorsed a growth mindset reported less maladaptive coping, which was in turn related to higher resilience during the pandemic. This is a complete mediation, as the direct relationship (path *c’*) between growth mindset and resilience was not significant, *t*(258) = −0.56, *p* = 0.579.

For acceptance coping, there was a significant path *a*, *t*(259) = 4.58, *p* < 0.001, a significant path *b*, *t*(258) = −2.57, *p* = 0.011, and a significant path *c*, *t*(259) = −2.05, *p* = 0.041. There was a significant indirect effect (path a*b) of growth mindset on resilience through acceptance coping, b = −0.048, Bootstrap CI_95_[−0.098, −0.005], meaning that students who endorsed a growth mindset reported more acceptance coping, which was in turn related to higher resilience during the pandemic. This is a complete mediation, as the direct relationship (path *c’*) between growth mindset and resilience was not significant, *t*(258) = −1.29, *p* = 0.199.

### Intervention effects

**Figure 4:**
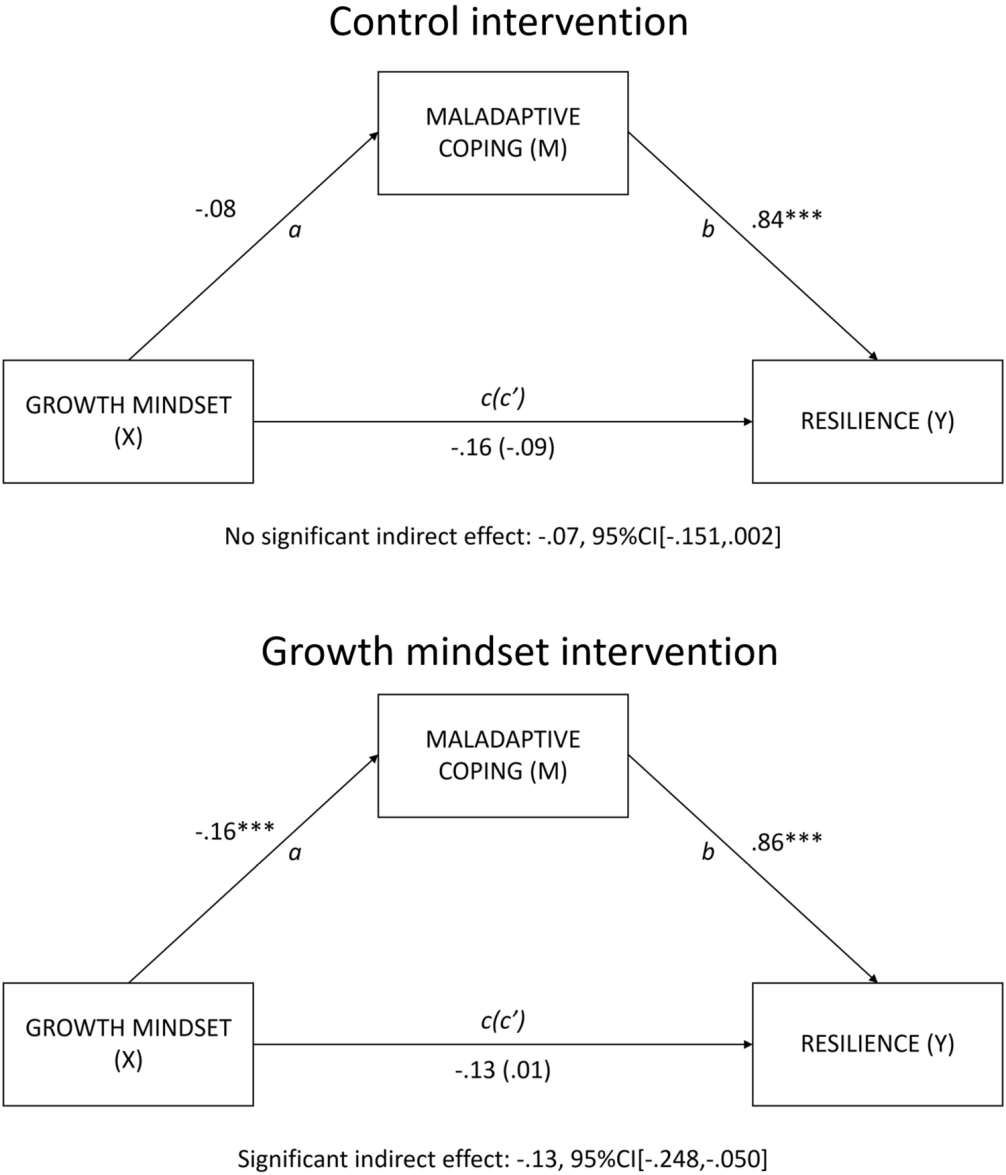
Mediation results: separately for control and growth mindset intervention groups. *Note. N (*control intervention group) = 130. *N (*growth mindset intervention group) = 131. X = independent variable. Y = dependent variable. M = mediator. The control and growth mindset interventions took place one year before the pandemic started (two years before the peri-pandemic measures). Note, growth mindset and maladaptive coping were measured during the pandemic (T3). The resilience variable was defined as residuals of the regression between school burnout symptoms and COVID-related stress exposure (at T3), controlling for pre-pandemic school burnout symptoms (T2). Note that the resilience variable is reversed. * p < .05, ** < .01, *** < .001; path c’ = direct effect; numbers are beta values.

Maladaptive coping significantly mediated the relation between growth mindset and resilience, only for the growth mindset intervention group, but not the control intervention group, see Fig. 4 (exploratory hypothesis 3). For those who received the growth mindset intervention two years prior, there was a significant path *a*, *t*(129) = −4.00, *p* < 0.001, a significant path *b*, *t*(128) = 4.15, *p* < 0.001, but not a significant path *c*, *t*(129) = −1.30, *p* = 0.196. There was a significant indirect effect (path a*b) of growth mindset on resilience through maladaptive coping, b = −0.135, Bootstrap CI_95_[−0.248, −0.050], meaning that students who endorsed a growth mindset reported less maladaptive coping, which was in turn related to higher resilience during the pandemic.

## Discussion

The COVID-19 pandemic impacted adolescent mental health on a global scale^1^. At the same time, many students were resilient during this crisis, despite exposure to COVID-related stressors. These students may be protected against school-related mental health consequences, due to predispositions (‘resilience factors’) that make resilient responding to a stressor more likely^16,17^. We aimed to study the protective effect of growth mindset on school-related resilience during the COVID-19 pandemic, and the mediating effects of coping styles. We addressed several limitations of previous studies, by (1) including a measure of school-related mental health (school burnout symptoms) before and during the pandemic, (2) manipulating growth mindset *before* the pandemic with an RCT design and follow-up during the pandemic, (3) operationalizing school-related resilience as an outcome, in line with the definition of ‘maintaining mental health despite stressor exposure’, taking into account the actual exposure to COVID-19-related adversity, (4) investigating the role of coping styles, which can provide a mechanistic understanding of how mindset affects school-related resilience.

First, we hypothesized that students with a growth mindset would (1a) demonstrate more school-related resilience and, (1b) use more adaptive coping strategies. To test the resilience hypothesis (1a), we first needed to regress mental health (school burnout) on stressor exposure, while correcting for pre-pandemic mental health. In line with national and international research^1,6,11,12^, students who were exposed to more COVID-related stressors, reported deteriorated school-related mental health in the current study. Subsequently, we quantified resilience as the residuals onto this regression curve—i.e., a more resilient student would report better mental health than expected, given the stressor exposure, and thus have a negative residual. In line with our hypothesis, students who more strongly endorsed a growth mindset were more resilient during the pandemic.

To test the coping hypothesis (1b), we first identified six higher-order coping styles with principal component analysis: maladaptive (*behavioral disengagement, self-blame, venting*), active (*active coping, planning, positive reframing, self-distraction*), social support, religion, acceptance and humor. Although the literature is inconclusive with regards to the categorization of coping styles^30^, most items in our maladaptive coping factor are consistent with the literature (either named maladaptive coping, avoidance coping, or evasive coping)^32^, and in line with the originally theorized ‘less useful’ coping^55^. The maladaptive nature of our factor was also confirmed by our findings, as it was related to less resilience. Our active coping factor includes items previously categorized as approach, adaptive or active coping (in line with theorized problem-focused coping^55^), while other items were part of emotion-focused coping^32^. Surprisingly, this factor was not related to resilience according to our findings. The acceptance factor in our study may also be seen as adaptive, considering previous research^32^, and its relation with higher resilience in our study. The social support scale has been categorized as adaptive and socially supported coping in previous studies^32^. The religion and humor scales were identical to the original brief COPE scales^31^. The humor scale has been inconsistently considered as avoidance coping, positive coping, and emotion-focused coping^32^, but in our study it seems to be a maladaptive/avoidance form of humor, as it was related to less resilience. Finally, mostly in line with our hypotheses, students who more strongly endorsed a growth mindset, reported less maladaptive coping and more adaptive coping (acceptance factor). In addition, they reported less coping with humor.

Second, we hypothesized that the positive association between growth mindset and resilience would be mediated by coping styles. We confirmed this hypothesis. Students who endorsed a growth mindset reported less maladaptive coping/more adaptive (acceptance) coping, which were in turn related to higher resilience during the pandemic. Third, when we further explored the intervention subsamples, the mediation of maladaptive coping was only present for students who had received the growth mindset intervention two years earlier, but not for those who received the control intervention. Overall, our findings fit with theoretical predictions of how beliefs form a framework for assigning meaning to events, especially when facing adversity^22^, such as the COVID-19 pandemic, and corroborate evidence of protective effects of growth mindset on mental health during^18,27,28^ and before^20,25^ the pandemic.

In this study we confirmed that growth mindset is a resilience factor, but importantly, also one that can be trained. We addressed an important gap in the literature, which was identified by Burnette et al.^20^; studies that investigated links between mindsets and coping rarely, if ever, manipulated mindsets. Maladaptive coping mediated the relation between mindset and resilience, only in the growth mindset intervention group. As we hypothesized that coping is the mechanism that explains how mindset affects mental health in schools, the mediation suggests that this mechanism may only have been activated in students who had received the growth mindset intervention before the pandemic. Interestingly, the results of the one-year follow-up of our growth mindset intervention study did not show beneficial effects on school burnout symptoms^39^, while the current two-year follow-up demonstrated this mediation. This discrepancy may be explained by the important role of set-backs in amplifying differences between growth and fixed mindset^23^. Only the two-year follow-up was characterized by such a massive setback, in the form of COVID-19.

Active coping was not related to mindset and resilience. A possible reason may be sought in the type of stressor that COVID-19 represented. Although students could control to a certain level how strictly they followed lockdown rules and the risk for infection, to a large extent there were external consequences at play outside students’ control, including lockdown measures that disproportionally affected schools. In turn, this may have induced an external locus of control, in which active coping strategies were less applicable. This seems to be supported by the fact that acceptance coping was related to mindset and resilience, which involved ‘accepting the reality’ of the pandemic. In addition, developmental changes in coping during adolescence^56^ may explain why we could not demonstrate effects for active coping. Adolescents use cognitive coping styles to a lesser extent than adults^57^, and younger adolescents use less active coping styles (e.g., planful problem solving; reappraisal) compared to older adolescents^58^.

A few limitations of the current study should be considered. First, previous work shows that the strength of associations between mindset and psychological distress and coping are dependent on mindset domain, with stronger effects for emotion-based mindsets, and smaller but significant effects for intelligence-based mindsets^20^. The current study focused on the latter, which is more common in the school context, and maybe more relevant in combination with school burnout symptoms as outcome measure. However, it remains unknown whether emotion-based mindsets would have been a better or additional predictor of coping and school burnout symptoms. Related to that, these results cannot be generalized outside the school context. Second, although they were obtained simultaneously, the questionnaires referred to the last year (COVID-related stress exposure and coping), or the time period was not specified (school burnout symptoms, mindset), which reduces the temporal specificity/alignment of the results. Third, it remains unknown whether growth mindset is a unique resilience factor, and how it relates to other resilience factors, such as positive appraisal style^17^. Related to this, other ‘adaptive systems’ that support student’s resilience, such as social connections and community services, are promising avenues to further explore^59^, in addition to potential interactions with personality characteristics^60,61^. Fourth, the separate mediation analyses for the control and growth mindset intervention groups were exploratory, as they were not originally planned when we designed and registered this RCT. Lastly, most effects were small to moderate, and discussions about what constitutes a meaningful effect size^62^ are applicable here as well.

The present work also has considerable strengths. Due to its unique and coincidental timing, we could control for pre-pandemic school burnout symptoms, which contributes to the specificity of our findings concerning school-related resilience during COVID. In addition, the RCT design allowed us to explore whether a growth mindset intervention influenced associations between mindset, coping and resilience two years later, indicating tentative causal evidence. In addition, we considered resilience as an outcome measure—maintaining mental health despite stressor exposure—which we operationalized based on individuals’ residuals onto the regression curve between mental health (school burnout symptoms) and stress exposure (based on^36^), while correcting for pre-pandemic mental health. We used a scoring scheme to arrive at a more objective measure of exposure^17^, with minimal appraisal.

In conclusion, we presented unique evidence for the beneficial effects of growth mindset on school-related resilience during the COVID-19 pandemic, and the mediating effects of coping styles as explanatory mechanism. This work contributes to a growing literature that shows positive effects of growth mindset on mental health^18,20^, and adds to the resilience literature, by identifying a promising resilience factor that may be considered in futures studies.

## Supplementary Information

Supplementary Information 1.

Supplementary Information 2.

## Data Availability

This study’s primary design and hypotheses were preregistered; see https://www.trialregister.nl/trial/7562. Materials have been made publicly available at DataverseNL and can be accessed at https://doi.org/10.34894/EULELM. Data and analysis code for this study are available by emailing the corresponding author.
