# Supplementary material for: Coping styles mediate the relation between mindset and academic resilience in adolescents during the COVID-19 pandemic: a randomized controlled trial
Source: Sci Rep. 2023 Apr 13;13:6060. doi: 10.1038/s41598-023-33392-9 (PMC10099024; doi:10.1038/s41598-023-33392-9)
Supplement: Supplementary file 2 — Supplementary Information 2. [file 41598_2023_33392_MOESM2_ESM.docx]

**Supplementary Tables S2 and S3**

The following results describe the PCA to identify higher-order coping styles, based on 26 items of the brief COPE.

**Supplementary Table S2**

| **Pattern Matrix** | | | | | | | |
| --- | --- | --- | --- | --- | --- | --- | --- |
|  | Component | | | | | | |
|  | 1 | 2 | 3 | 4 | 5 | 6 |  |
| **C_2_2** | .784 |  |  |  |  |  |  |
| **C_2_12** | .765 |  |  |  |  |  |  |
| **C_1_13** | .695 |  |  |  |  |  |  |
| **C_1_6** | .673 |  |  |  |  |  |  |
| **C_1_9** | .403 |  |  |  |  |  |  |
| **C_1_7** |  | .693 |  |  |  |  |  |
| **C_1_2** |  | .689 |  |  |  |  |  |
| **C_2_3** |  | .649 |  |  |  |  |  |
| **C_1_12** |  | .640 |  |  |  |  |  |
| **C_1_14** |  | .608 |  |  |  |  |  |
| **C_1_1** |  | .518 |  |  |  |  |  |
| **C_2_11** |  | .511 |  |  |  |  |  |
| **C_2_5** |  | .433 |  |  |  |  |  |
| **C_1_5** |  |  | -.881 |  |  |  |  |
| **C_2_1** |  |  | -.869 |  |  |  |  |
| **C_1_10** |  |  | -.813 |  |  |  |  |
| **C_2_9** |  |  | -.676 |  |  |  |  |
| **C_2_7** |  |  |  |  |  |  |  |
| **C_2_13** |  |  |  | -.874 |  |  |  |
| **C_2_8** |  |  |  | -.843 |  |  |  |
| **C_2_6** |  |  |  |  | .841 |  |  |
| **C_2_10** |  |  |  |  | .752 |  |  |
| **C_1_8** |  |  |  |  | -.465 |  |  |
| **C_1_3** |  |  |  |  | -.415 |  |  |
| **C_2_4** |  |  |  |  |  | -.829 |  |
| **C_2_14** |  |  |  |  |  | -.770 |  |

Note. Factor loadings

**Table next page: Supplementary Table S3**

Note. Overview of factors, proposed categories of coping, original scales and scale questions.

| **Factor** | **Proposed category** | **Original scale** | **Scale questions (Variable name)** |
| --- | --- | --- | --- |
| 1 | Maladaptive | Behavioral disengagement | I've been giving up trying to deal with it (C_1_6) |
|  |  | Venting | I've been saying things to let my unpleasant feelings escape (C_1_9) |
|  |  | Self-blame | I’ve been criticizing myself (C_1_13) |
|  |  | Behavioral disengagement | I've been giving up the attempt to cope (C_2_2) |
|  |  | Self-blame | I’ve been blaming myself for things that happened (C_2_12) |
| 2 | Active | Self-distraction | I've been turning to work or other activities to take my mind off things (C_1_1) |
|  |  | Active coping | I've been concentrating my efforts on doing something about the situation I'm in (C_1_2) |
|  |  | Active coping | I've been taking action to try to make the situation better (C_1_7) |
|  |  | Positive reframing | I've been trying to see it in a different light, to make it seem more positive (C_1_12) |
|  |  | Planning | I've been trying to come up with a strategy about what to do (C_1_14) |
|  |  | Positive reframing | I've been looking for something good in what is happening (C_2_3) |
|  |  | Self-distraction | I've been doing something to think about it less... (C_2_5) |
|  |  | Planning | I've been thinking hard about what steps to take (C_2_11) |
| 3 | Social support | Emotional support | I've been getting emotional support from others (C_1_5) |
|  |  | Use of informational support | I’ve been getting help and advice from other people (C_1_10) |
|  |  | Emotional support | I've been getting comfort and understanding from someone (C_2_1) |
|  |  | Use of informational support | I’ve been trying to get advice or help from other people about what to do (C_2_9) |
| 4 | Religion | Religion | I've been trying to find comfort in my religion or spiritual beliefs (C_2_8) |
|  |  | Religion | I've been praying or meditating (C_2_13) |
| 5 | Acceptance | Denial | I've been saying to myself "this isn't real" (C_1_3) > reversed scored, before calculating scale |
|  |  | Denial | I've been refusing to believe that it has happened (C_1_8) > reversed scored, before calculating scale |
|  |  | Acceptance | I've been accepting the reality of the fact that it has happened (C_2_6) |
|  |  | Acceptance | I've been learning to live with it (C_2_10) |
| 6 | Humor | Humor | I've been making jokes about it (C_2_4) |
|  |  | Humor | I've been making fun of the situation (C_2_14) |
| Excluded |  | Substance use | I've been using alcohol or other drugs to make myself feel better (C_1_4) |
|  |  | Substance use | I've been using alcohol or other drugs to help me get through it (C_1_11) |
| No loading |  | Venting | I've been expressing my negative feelings (C_2_7) |

**Rationale for interpretation/naming factors**

**(1) Maladaptive**

Previous studies (described in ^1^) categorized the two ‘behavioral disengagement’ items and the two ‘self-blame’ items consistently as *maladaptive coping, avoidance coping,* or *evasive coping*. In most studies, ‘venting’ is also part of one of those categorizations. All individual items, such as “I’ve been blaming myself for things that happened”, can be argued to be unhelpful or maladaptive. In addition, higher scores on this scale were related to less resilience in the current study, further confirming the interpretation and naming of this factor.

**(2) Active**

Previous studies (described in ^1^) categorized the two ‘active coping’ items and the two ‘planning’ items as *approach coping, adaptive coping,* or *active coping*. The two ‘positive reframing’ items have been categorized in there as well, but also as *emotion-focused coping*. The two ‘self-distraction’ items have both been categorized as *avoidant coping* and *adaptive coping*, which is less consistent. Although one could argue to name this factor ‘adaptive coping’, all items have in common that they involve active behaviors or cognitions, therefore we named this factor ‘Active’.

**(3) Social support**

Previous studies ^1^ categorized the two ‘emotional support’ items and the two ‘informational support’ items as *approach/adaptive coping* or *support/socially supported coping*. Considering that all items have support of others in common, we named this factor ‘social support’.

**(4) Religion**

These two items correspond to the original scale of the brief COPE.

**(5) Acceptance**

Previous studies ^1^ categorized the two ‘acceptance’ items as *approach coping, adaptive coping, positive coping* and *emotion-focused coping*. While the two ‘denial’ items are consistently categorized as *avoidance coping, maladaptive coping,* and *evasive coping*, they have a negative loading in our results. Considering that the two items (e.g., “I've been refusing to believe that it has happened”) are the opposite of acceptance, we decided to name this scale ‘acceptance’ (note that the two ‘denial’ items are reverse scored). Our results showed that more resilient students reported higher acceptance coping, which shows this coping style is adaptive.

**(6) Humor**

These two items correspond to the original scale of the brief COPE. Interestingly, in previous studies this scale has been inconsistently considered as *avoidance coping*, *positive coping,* and *emotion-focused* coping. This may show that humor sometimes is adaptive and sometimes maladaptive. Our results showed that more resilient students reported less coping with humor, which seems to indicate that this coping style is maladaptive in our population (maladaptive humor).

**References**

1. Hanfstingl, B. *et al.* The Dimensionality of the Brief COPE Before and During the COVID-19 Pandemic. *Assessment* 107319112110524 (2021). doi:10.1177/10731911211052483
